# Hunger & satiety signals: another key mechanism involved in the NAFLD pathway

**DOI:** 10.3389/fendo.2023.1213372

**Published:** 2023-09-11

**Authors:** Iván López-Méndez, Andrea Del Carmen Maldonado-Rojas, Misael Uribe, Eva Juárez-Hernández

**Affiliations:** ^1^Hepatology and Transplants Unit, Medica Sur Clinic & Foundation, Mexico City, Mexico; ^2^Translational Research Unit, Medica Sur Clinic & Foundation, Mexico City, Mexico; ^3^Gastroenterology and Obesity Unit, Medica Sur Clinic & Foundation, Mexico City, Mexico

**Keywords:** obesity, metabolic syndrome, hormones, peptides, energy intake

## Abstract

Non-alcoholic fatty liver disease (NAFLD) is a highly prevalent metabolic disease, although prevalence could change according to region, nowadays is considered a public health problem whose real impact on the health system is unknown. NAFLD has a multifactorial and complex pathophysiology, due to this, developing a unique and effective pharmacological treatment has not been successful in reverting or avoiding the progression of this liver disease. Even though NAFLD pathophysiology is known, all actual treatments are focused on modifying or regulating the metabolic pathways, some of which interplay with obesity. It has been known that impairments in hunger and satiety signals are associated with obesity, however, abnormalities in these signals in patients with NAFLD and obesity are not fully elucidated. To describe these mechanisms opens an additional option as a therapeutic target sharing metabolic pathways with NAFLD, therefore, this review aims to describe the hormones and peptides implicated in both hunger-satiety in NAFLD. It has been established that NAFLD pharmacological treatment cannot be focused on a single purpose; hence, identifying interplays that lead to adding or modifying current treatment options could also have an impact on another related outcome such as hunger or satiety signals.

## 1 Introduction

Nonalcoholic fatty liver disease (NAFLD) has become one of the most common chronic liver diseases in the world and its prevalence has increased along with the obesity pandemic (1). Current data indicates that NAFLD prevalence in the world population is around 25%, yet it might vary according to age, sex, and ethnicity (2).

NAFLD is associated with various metabolic comorbidities including obesity (51.34%; 95% CI: 41.38 - 61.20), type 2 diabetes (DM) (22.51%; 95% CI: 17.92 - 27.89), hyperlipidemia (69.16%; 95% CI: 49.91 - 83.46), hypertension (39.34%; 95% CI: 33.15 - 45.88), and metabolic syndrome (MetS) (42.54%; 95% CI: 30.06 - 56.05). Due to these complex associations, performing clinical trials in NAFLD patients without metabolic comorbidities is practically impossible; therefore, treatment options need to be effective for more than one metabolic outcome. Clinically, most NAFLD patients tend to be obese, with other characteristics of MetS, which are all risk factors for cardiovascular diseases, one of the leading causes of death in these patients (3). However, NAFLD can also be found in lean people, which has been characterized as the non-obese NAFLD or lean NAFLD (4). The presence of liver steatosis in these patients is more related to epigenetic regulation, environmental and genetic factors; however, fibrosis and cardiovascular mortality seem to be higher than in overweight patients; therefore, lean NAFLD patients are not completely benefited by weight loss. Strategies focused on physical activity and body composition have been associated with an improvement in clinical outcomes in lean NAFLD patients; however, pharmacological clinical trials do not include these group of patients (5).

The ingestion of the appropriate amount of each nutrient factor is essential for maintaining body homeostasis. Improper nutritional balance has been linked to a wide spectrum of disorders; for example, overeating leads to obesity (6). Since NAFLD is majorly associated with obesity and its complications, one of the mechanisms implicated in the development of this liver disease is the imbalance between hunger and satiety. Hunger and satiety sensations are modulated by an interplay between the gastrointestinal tract and the brain, which control the amount of food ingested during a meal, and the hunger or satiety sensations related to alterations of energy balance and expenditure (7).

Hunger and satiety are modulated majorly by hormones and peptides. Ghrelin (Ghr), thyroid hormones (TH), glucocorticoids (GC), and melanin-concentrating hormone (MCH) are the hormones implicated in hunger, while orexin, neuropeptide Y (NPY), and opioid peptide are the peptides that have been associated with this process. On the other hand, glucagon like peptide-1 (GLP-1) and peptide YY (PPY) have been associated with satiety; cholecystokinin (CCK), somatostatin (SST), and leptin are the hormones involved in this sensation (6, 8). The aim of this review is to expose the mechanisms of action of the most important hormones and peptides involved in hunger and satiety, as well as their role in NAFLD development.

## 2 Hunger

Food intake control is regulated by peptidergic regulators stimulating hunger to initiate food intake. In patients with metabolic or weight abnormalities, alterations in the interaction of peripheral and central signals could be related to impaired energy homeostasis and food intake regulation (9). Hunger is defined as a craving or urgent need for food (10). Most perceived hunger signals originate in the stomach, where the vagus nerve generates an electric signal when there is a state of emptiness, reinforced by Ghr secretion and by the metabolic signal of hypoglycemia. The accumulation of excess body fat results when energy intake exceeds energy expenditure; despite the fact that energy balance and food intake are controlled by hypothalamic responses, it has been observed that these can be overridden by hedonic reward brain systems, leading to food consumption beyond homeostatic needs (11).

There are several hormones and peptides affecting brain centers involved in the central control of hunger and satiety. Peptides act in two pathways that are intertwined by neural networks: one in the arcuate nucleus of the hypothalamus and the other one in the solitary tract (9). On the other hand, hormones are involved in pathways of obesity, but it has been observed that there are specific effects of hormones in NAFLD itself; however, this role is complex and needs to be differentiated from the pathways involved in obesity (12). Regarding hunger regulation, the involvement of a wide variety of hormones and peptides related to obesity and NAFLD could be implicated in NAFLD pathophysiology in obese patients.

### 2.1 Ghrelin

Ghr is produced by gut endocrine cells mostly located in the gastric oxyntic glands, in the fundus of the stomach (13). The Ghr effect on feeding regulation occurs in the hypothalamus; growth hormone-releasing peptides and growth hormone secretagogues stimulate food intake by activating hypothalamic neurons involved in homeostatic feeding regulation (14). The most prominent effect of Ghr is its ability to stimulate food intake through an increase in the activity of neurons expressing neuropeptide and agouti-related protein (AgRP) (15). Also, Ghr is involved in the regulation of lipid metabolism; therefore, alterations in its expression have an effect in fat distribution and mobilization, leading patients with Ghr deficiency to present increased lipid levels and body weight (BW), as well as body composition abnormalities (16).

Ghr level increases during dieting and this could explain the difficulty for achieving long-term results from dieting (17). In liver disease associated with obesity, the liver damage signal is sent to the brain and stomach via autonomic nerve connections, which causes an increased Ghr release. These signals could slow down the progression of NAFLD (18), which makes the Ghr o-acyltransferase an interesting pharmacological target (19). A hypothesis that Ghr might be involved in NAFLD was proposed by Liu et al. using a murine NAFLD model divided into control and NAFLD groups; they observed that a plasma decrease in Unacylated Ghr/Acylated Ghr ratio in combination with the hypothalamic over-expression of Acylated Ghr and its receptor could be associated with NAFLD due to their positive correlations (R2 = 0.56 – 0.85, p <0.05) with homeostatic model assessment insulin resistance (HOMA-IR) (20).

A cross-sectional study with 91 DM patients with and without NAFLD found the serum acyl-Ghr as a diagnostic marker for NAFLD (21). The results showed 1.5 (p = 0.016) and 2.5 (p ≤0.001) fold increase of serum acyl-Ghr levels in patients with NAFLD and normal or elevated transaminases compared with control groups (OR 1.791; 95% CI 1.162 – 2.759; p = 0.008). Therefore, serum acyl-Ghr was evaluated as a non-invasive marker for NAFLD detection with diagnostic accuracy of AUROC 0.835 (95% CI 0.752 – 0.918, p <0.001), with a cut-off value of >0.52 ng/ml. Acyl-Ghr was less effective for distinguishing between patients with NAFLD and elevated transaminases and patients with NAFLD and normal values.

The effects of Ghr and its receptor antagonist on BW loss, reducing food intake, and adiposity through the reduction of appetite and augmentation of energy expenditure and fat catabolism have been studied and proposed as a therapeutic option (22). Pegvisomant is a Ghr antagonist that has been observed to bind to the Ghr at the cell surface and hence to block this process; yet further investigation is needed to conclude if this molecule is able to change the course of NAFLD (23).

### 2.2 Thyroid hormones

It has been observed that the thyroid axis regulates feeding and responds to changes in the nutritional status. Hypothalamic thyrotropin-releasing hormone expression has been related to effects of fasting, down-regulating the hypothalamic-pituitary-thyroid axis and in with the effect of decreasing energy expenditure; on the other hand, the direct anorectic effect of this hormone may regulate food intake, independently of its effects on the hypothalamic-pituitary-thyroid axis (24).

The relationship between thyroid status and obesity is likely to be bidirectional, with hypothyroidism affecting weight and body mass index and obesity also influencing thyroid function (25). On the other hand, the liver and the thyroid are intimately linked, with TH playing important roles in *de novo* lipogenesis, β-oxidation, cholesterol metabolism, and carbohydrate metabolism (26). TH stimulates lipolysis from fat stores in the white adipose tissue and from dietary fat sources to generate circulating free fatty acids (FFA), whereas hypothyroidism increases triglyceride-derived fatty acid uptake in the white adipose tissue and decreases its uptake in the liver (27).

Regarding patients with thyroid dysfunction, a negative relationship with Ghr and THs has been proposed due to their effects on the hypothalamic-pituitary-adrenal axis. In a large-scale study with 1012 patients, no associations were found between serum levels of Ghr and thyroid stimulating hormone (28). Recent studies in rodents and patients further support this inverse relationship between physiological thyroid status and NAFLD. Triiodothyronine administration significantly decreases liver steatosis and inflammation, also restoring the mitochondrial function in non-alcoholic steatohepatitis (NASH) (29).

THs and their metabolites, along with the TH receptor-β agonist and other liver specific analogs, have been tested in the last few years as a potential NAFLD therapy (30). The most promising is Resmetirom, a selective TH Receptor-β agonist; Harrison et al. (31) reported beneficial effects of this molecule in a randomized clinical trial controlled with placebo in patients with NASH; they observed a significant reduction of hepatic fat after 12 (−32·9% Resmetirom *vs* −10·4% placebo) and 36 (−37·3% Resmetirom [n = 74] *vs* −8·5 placebo [n = 34]) weeks of treatment with Resmetirom compared with placebo. Despite these positive results, more studies that evaluate histological effects or changes in non-invasive markers are needed to recommend TH Receptor-β agonists as treatment for NAFLD patients.

### 2.3 Glucocorticoids

Glucocorticoids (GC) are endogenous adrenal steroid hormones produced in the adrenal glands in response to stress. The excessive production of GC leads to a severe metabolic dysfunction characterized by obesity, insulin resistance (IR), hyperglycemia, muscle wasting, and liver steatosis (32). Most of the effects of this high production occur upon binding to the GC receptor, so a partial or generalized GC resistance syndrome may result from a reduced level of functional activity of the GC receptor and a decreased hormone affinity and binding (33).

Specifically, cortisol, the principal GC in humans that increases in response to stress, has been related to appetite ratings and energy intake; salivary cortisol has been associated with increased hunger and energy intake, as well as modulation of leptin, NPY, and cytokines in response to stress. Thereby, cortisol has been proposed as a biomarker of appetite (34, 35).

Even if GC have been associated as regulators in food intake and energy expenditure strongly related with obesity, the mechanism that contributes to GC inducing NAFLD has not been explored in detail in clinical studies. However, GC have been observed to play a key role in fatty acids increase and lipogenesis decrease, in absence of insulin, both in *in vitro* and animal s models (36). On the other hand, the loss of GC receptor signaling is associated with alterations in adiponectin and leptin expressions (37). This loss has been observed in high fat diets (HFD) and high adipose tissue, but its impact on NAFLD was not assessed. Increased circulating GC levels have been suggested to be an important driver of the disease (38).

In a study in mice, Koorneef et al. observed the efficacy of CORT118335, a selective glucocorticoid receptor modulator, in the reduction of hepatic lipid accumulation, in animal models; after HFD, a prevented and reversed hepatic lipid accumulation was observed in those animals treated with COT118335. The authors concluded that this effect could be explained by the stimulation efflux of very low-density lipoproteins and the lack of stimulation of fatty acids uptake, both dependent of GC receptors. These results suggest that CORT118335 could be a potential treatment for NAFLD (39).

### 2.4 Orexin

Orexin peptides (also known as hypocretins), comprised by two isoforms named orexin-A/hypocretin 1 and orexin-B/hypocretin 2, are produced in the lateral hypothalamus and adjacent regions, and then released widely in the central nervous system (40). These two neuropeptides regulate BW, glucose homeostasis, insulin sensitivity, and the hunger-satiety cycle (41). Orexin-A and its receptor have been implicated more strongly in the regulation of feeding behavior; this peptide is released in the brain and the gut, increasing food intake and energy expenditure simultaneously (42).

The role of orexin in non-alcoholic steatohepatitis (NASH) development has been studied by Tsuneki et al; they observed a severe obesity, with NASH and fibrosis progression, in orexin-deficient mice; additionally, when mice were long-term fed with HFD, they showed a progression to hepatocellular carcinoma (HCC). After an intervention with daily supplementation of orexin A, through intracerebroventricular injection, an attenuation of inflammation and hepatic endoplasmic stress was observed, concluding that adequate levels of orexin A could prevent NASH and HCC progression (43). However, this research was carried out in mice and current literature shows no studies in humans. Further research in clinical trials with humans is needed to determine if orexin is an adequate preventative therapy for NASH.

### 2.5 Neuropeptide Y

NPY is expressed in many neuron types, promoting attraction to food through olfactory cues (44, 45). NPY is known as an orexigenic peptide with effect in adipocytes; Park et al. observed in a deficient NPY mice model, fed with HFD, a significant decrease in BW, adiposity, liver steatosis, and adipose inflammation compared to NPY +/+ mice with a standard diet (46). On the other hand, in a rat model injected with NPY in the hepatic portal vein, Chen et al. observed that hepatic cholesterol content increased after NPY injection within just 1 h of treatment. An effect of NPY seems to occur in the activation of the cholesterogenic pathway in hepatocytes (47). Even though NPY has been related with lipid and adiposity abnormalities, which have a key role in NAFLD development, the mechanism of the regulatory effects of NPY is still unknown; however, an NPY antagonist could be a novel target in pharmacological options for obesity and NAFLD.

### 2.6 Melanin-concentrating hormone

MCH is a neuropeptide produced in the hypothalamic area implicated in food intake and BW gain (48). It has been observed that the lack of an MCH or MCH1 receptor could have anti-obesity effects; therefore, it has been suggested as a potential target in obesity and NAFLD (49). Kawata et al. showed that the administration of an MCH1 receptor antagonist decreased BW and had an anorectic effect in a murine model; it also reduced lipid content and the expression level of genes implicated in lipogenesis, inflammation, and fibrosis (50).

Despite the HFD, MCH-deficient mice are lean, hypophagic, and do not develop liver steatosis; in contrast, NAFLD is promoted when MCH receptors are activated, with increasing fat deposition independent of food intake and energy expenditure. MCH controls lipid accumulation, hepatic lipid uptake, lipid storage, and the decrease in lipid mobilization in adipocytes (51), being an interesting target for appetite suppression treatments; however, developing an MCH-receptor 1 antagonist is challenging due to its risk of cardiotoxicity. Lim et al. identified KRX-104130, which has a potent MCH-receptor 1 antagonistic activity and no cardiotoxicity. In a NASH model, the administration of this antagonist showed a significant decrease in liver triglycerides (TG) levels, as well as in serum concentrations of aspartate amino transferase (AST) and alanine aminotransferase (ALT); additionally, they observed an improvement in steatosis and fibrosis in histopathological analysis (49). These results support the potential therapeutic effect of MCH-receptor 1 antagonists as a future option for NAFLD patients.

**Figure 1:**
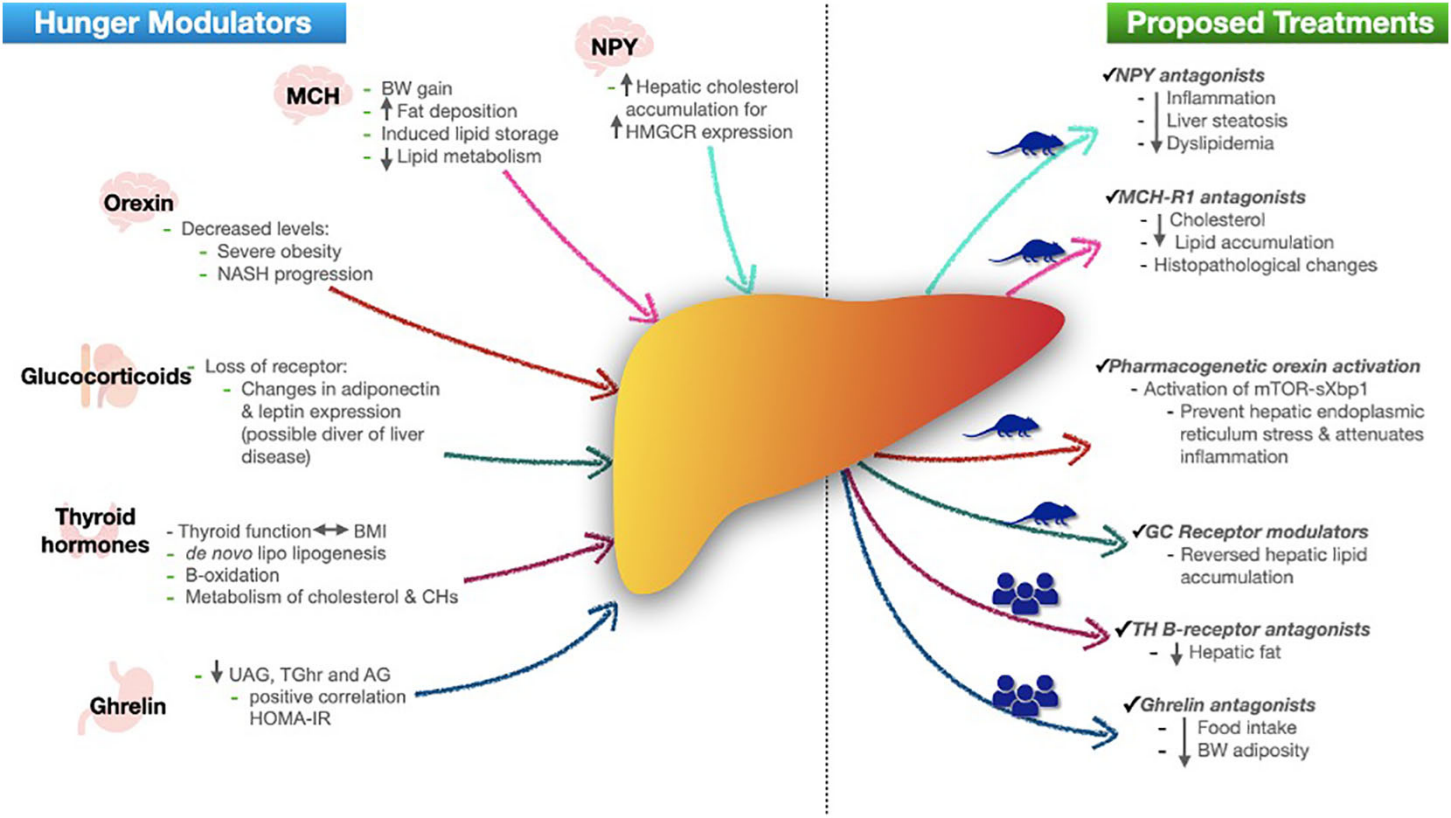
Role of hunger modulators in NAFLD and proposed treatments. In left side, figure shows each hunger modulator (with their secretion site) role in development of NAFLD. In right side shows the actual proposed treatments based in human or mice models. NPY neuropeptide Y; HMGCR 3-hydroxy-3-methylglutaryl-CoA reductase; MCH melanin-concentrating hormone; BW body weight; TG triglycerides; NASH non-alcoholic steatohepatitis; BMI body mass index; CHs carbohydrates; UAG unacylated ghrelin; TGhr total ghrelin; AG acylated ghrelin; HOMA-IR homeostatic model assessment insulin resistance; MCH-R1 melanin-concentrating hormone receptor-1; GC glucocorticoids; TH thyroid hormones.

There is evidence about the relationship of hunger modulators with NAFLD pathways, therefore, some adds, or modifications of these agents have been proposed as a treatment, majorly in animal models (**Figure 1**).

## 3 Satiety

Further eating inhibited by “fullness” is termed satiety (11). Peptide signals act to optimize the digestive process and as short-term satiety signals. Their main actions include delaying gastric emptying, stimulating pancreatic enzyme secretion, and stimulating gall bladder contraction (52). The satiety cascade can be explained on three related levels: 1) physiological and behavioral patterns, 2) peripheral physiological and metabolic events, 3) neural and metabolic interactions in the brain. Sensory information derived from the cephalic phase of digestion and the afferent vagal signaling elicited by the presence of food in the stomach provides early information to the brain concerning the amount and nutrient content of the food consumed (53).

The disruption in the balance of satiety signals induces an imbalance between energy intake and energy expenditure, leading to either weight gain or weight loss (7); therefore, we will discuss the role of anorexigenic hormones that increase satiety and are involved in several physiological processes in the body, such as regulation of energy homeostasis, body fat accumulation, regulation of appetite, energy balance, and BW, since reduced satiation can lead to obesity, which brings along its complications including hypertension, DM, obstructive sleep apnea, and NAFLD (54). Understanding the role of satiety in NAFLD pathophysiology could provide strategies or treatment options for these patients, based on improving the satiety sensation and preventing those trigger factors of NAFLD, such as obesity and body fat accumulation.

### 3.1 Cholecystokinin

Cholecystokinin (CCK) is a “gut-brain” peptide hormone, secreted by enteroendocrine cells which are sensors of nutrients and with a key role as metabolic signal transduction units. Eight subsets of enteroendocrine cells have been observed and CCK is expressed in five of them; therefore, there are different types of CKK classified according to molecular size, and it could be found in plasma (CCK-33) and brain (CCK-8) (55–57). High secretion of CCK depends on macronutrient (faster and longer in fats and proteins) and fiber content of meals. Proteins are also involved in the stimulation of CCK release via the inhibition of trypsin-mediated digestion of intestinal CCK releasing peptides (52).

The effect of CCK on appetite is mediated by CCK receptors type 1, which are present on vagal afferent neurons within the upper gut wall, controlling appetite in peripheric manner, and CCK receptors type 2, which are present in parietal cells and regulate gastric acid secretion together with gastrin (58). The CCK stimulation mediated by these receptors reduces food intake and gastric emptying by acting in a paracrine manner. Additionally, CCK controls the expression of two G-protein coupled receptors: GPR40 (FFAR1) and calcium-sensing receptors (CASR); therefore, at low plasma concentrations of CCK, vagal afferent neurons show an increased capacity for appetite-stimulation, while post-prandial concentrations of CCK lead to an increased capacity for satiety signaling (59, 60).

In a NASH prevention study and a reversal study, mice were supplemented with proglumide, a CCK receptor antagonist. Treatment with proglumide has been observed to prevent NASH and reverse altered liver transaminases with significant cellular changes: inflammation was reduced by 42% (p = 0.023), fibrosis was reduced by 28.6% (p = 0.037), and steatosis was reduced by 57% (p = 0.016) (61).

CCK has an apparent potential as adjunct therapy of GLP-1 based drugs, as well as with leptin and amylin inhibitors (62). This synergy could represent a beneficial strategy in the treatment of obesity and DM; however, more evidence, specifically in liver markers, is necessary to elucidate if this strategy is effective for NAFLD as well.

### 3.2 Glucagon-like peptide 1

GLP-1 is synthesized in L cells in the intestinal mucosa, a-cells in the pancreatic islet, and neurons in the nucleus of the solitary tract. Its plasma concentrations increase after food intake, acting as a hormone with different metabolic effects such as insulin secretion and decrease of gastric emptying (63, 64). GLP-1 in the gastrointestinal tract slows gastric emptying after a meal, promoting a feeling of fullness and a reduction of appetite; also its receptors on hypothalamic neurons stimulate satiety (65). It has been seen that the mechanism of satiety of GPL-1 is due to its effects on the central nervous system; GLP-1 receptors are present in the nucleus of the solitary tract and arcuate region, where they receive afferent input from the vagus and glossopharyngeal nerves and integrate both neural and humoral factors (66).

GLP-1 receptor agonists have a role in the activation of cyclic adenosine monophosphate, which stimulates the epidermal growth factor receptor, guiding to the activation of the PI3K/Akt signaling pathway that leads to suppress the expression of genes that have a role in the stimulation of insulin secretion, having an impact on the repression of hepatic gluconeogenesis and lipogenesis (67). They improve metabolic inflammation in the liver by suppressing the expression of profibrotic mediators, such as tumor growth factor-β, through the stimulation of cyclic adenosine monophosphate and inflammatory genes like TNF-α, IL-6, and nuclear factor NF-kappa-β (67–69). Also, the increased secretion of insulin and the improvement in glucose tolerance by GLP-1 agonist lead to a decreased lipogenesis *de novo* and enhance hepatic fatty acids oxidation and lipid export (18).

In Verdich et al. meta-analysis about the effect of GLP-1 infusion in human subjects, an average reduction of 11.7% in calorie intake was demonstrated. This reduction is dose-dependent and is not different between obese and lean patients (70). GLP-1 agonists and analogs can be integrated into the management of patients with DM, obesity, and NAFLD. Their effects appear to be largely mediated by delayed gastric emptying (71).

Liraglutide and semaglutide are the most studied GLP-1 receptor agonists R1-C14 for the treatment of metabolic abnormalities. Liraglutide has been studied as adjuvant therapy in weight regain for patients who underwent bariatric surgery (BS) (72); specifically in NAFLD patients, in the LEAN study, liraglutide showed resolution of steatosis, as well as metabolic improvement when it was compared with placebo (73). Semaglutide is the GLP-1 receptor agonist that has shown a clinically relevant reduction of BW in overweight or obese patients treated with a once-weekly 2.4 mg dose of Semaglutide in combination with lifestyle interventions (74). When Semaglutide was evaluated in NASH patients, Newsome et al. observed a significantly higher percentage of patients with NASH resolution than placebo (p <0.001 for Semaglutide 0.4 mg *vs*. placebo) (75, 76).

Both drugs have demonstrated to improve weight loss and metabolic markers in different types of patients, such as MetS patients and overweight/obese patients with and without DM, showing good tolerance; however, neither is free of adverse effects, majorly gastrointestinal symptoms (diarrhea, nausea, vomiting) and reactions in the application area (when injections are required). Although these symptoms are classified as mild to moderate and transitory, they could difficult patient adherence to these pharmacotherapies (77, 78).

Nowadays, Tirzepatide, a molecule with combined agonist action on GLP1 and glucose-dependent insulinotropic peptide, has demonstrated effectiveness in weight loss and improvement in metabolic parameters (especially glucose control), as well as in NAFLD markers. This molecule could be a new therapeutic option; however, the effects of Tirzepatide in satiety mechanisms are still unknown (79).

Obesity and DM are closely related with NAFLD. Due to the heterogeneity of NAFLD pathophysiology, the benefits of the GLP-1 receptor agonist on metabolic markers have an important impact in liver outcomes, such as liver function test, steatosis, and NASH improvement; nonetheless, more evidence is necessary to assess the effects of these drugs in fibrosis resolution (80, 81).

### 3.3 Peptide YY

Many peptides are synthesized and released by the gastrointestinal tract; one of them is PYY. There are two types of PPY: PPY 1-36 is postprandially released for intestinal L cells; once released, PPY 1-36 is cleaved at PPY3-36, which is a Y2 receptor agonist (82). Its role in gastrointestinal function regulation has been known for some time and it also influences eating behavior (66). Higher fasting PYY and greater postprandial secretion are noted in non-obese individuals compared with obese individuals, suggesting that a reduced feedback from PYY might be a key mechanism for obesity (83). In general, PYY is released into the circulation in response to a meal, in proportion to the calories ingested. Its secretion significantly delays gastric emptying and pancreatic secretion; it also has direct effects on adipocytes, reducing lipolysis *in vitro* (84). After PYY plasma levels are higher compared with meals consisting of protein and carbohydrates (66, 85).

There are many hypotheses about the action of PYY, but the most important is that it acts at the hypothalamus via vagal pathways afferent to the nucleus of the solitary tract, having its effect by the excitement of the arcuate pro-opiomelanocortin neurons and anorexigenic circuits activation (86). It has been seen that patients with reduced postprandial PYY release have lower satiety rates; the lack of PYY in mice results in hyperphagia and obesity (85). Finn et al. (87) recently analyzed the effect of a Takeda G protein-coupled receptor 5 agonist in a NAFLD and mild IR mice model, in combination with a 10mg/kg dose of linagliptin. They observed that this agonist induced PYY secretion with improvement in liver/body weight, as well as in liver triglycerides and cholesterol content (p <0.0001), indicating that inducing secretion of PYY, might have a therapeutic potential in patients with NAFLD; however, even with the partial intestinal absorption that this agonist has, the adverse effect related to gallbladder emptying with potential effect in bile acids accumulation could be still present; therefore, the interplays with gut microbiota and another intestinal effects, such as this partial absorption, should be considered confounding factors.

### 3.4 Somatostatin

SST is a growth hormone inhibitory peptide than acts as an endocrine hormone and as a local regulator, and also as a neurotransmitter and neuromodulator. SST is widely distributed in the central nervous system and other peripheral tissues, including pituitary, pancreas, thyroid, and the gastrointestinal tract (88). The mechanism of satiety that SST induces is linked to the reduction of food by a mechanism mediated by the vagal pathway, prolonging the intestinal transit time and interfering with meal absorption (89–91).

SST analogues, such as octreotide, lanreotide, vapreotide, and pasireotide, are more resistant to endogenous peptidases (92). In a case-control study, Li et al. investigated the expression levels of important regulators of the hepatic lipid metabolism and their possible effects of octreotide. They showed that octreotide treatment decreases fasting plasma glucose, insulin, TG, total cholesterol, low-density lipoprotein cholesterol, and FFA serum levels. The expression of hepatic proteins for fatty acid synthesis, such as sterol regulating binding protein 1c, was also reduced. This protein is an activated transcription factor that promotes the expression of enzymes involved in hepatic lipogenesis. Octreotide plays an important role in hepatic lipogenesis decrease and improves hepatic steatosis by increasing TG export from hepatocytes (93). Another study showed that hepatic TG and FFA levels were significantly decreased in patients treated with octreotide (TG 18.11 ± 7.08 p <0.01 *vs* the HFD group and FFA 40.86 ± 5.09 p <0.01 *vs* the HFD group). In this obesity-induced mice model, octreotide has been observed to improve hepatic glycogenesis and to decrease HOMA-IR and fasting glucose levels; however, changes in serum lipids and in liver function tests were not observed (94). Although octreotide could have beneficial effects on liver markers, its association with satiety signals and other metabolic markers needs to be extensively studied in order to propose octreotide or another SST analogue as multi-outcome treatment options.

### 3.5 Leptin

Leptin is a 16 kDa non-glycosylated hormone produced by adipocytes in proportion to fat size stores (95). Leptin also exerts an anti-steatotic effect, decreasing lipid accumulation and lipotoxicity, and a pro-inflammatory effect as well, stimulating fibrogenesis (96).

Animal models have shown that leptin prevents lipid accumulation specifically in the liver and in non-adipose tissues by lowering the expression of sterol regulating binding protein 1c (97); it has also been demonstrated that leptin prevents fatty liver through the activation of adenosine monophosphate protein kinase in the liver (98). However, obese patients generally present NAFLD despite their elevated leptin levels, which has led to think about different mechanisms of hepatic leptin resistance (99), such as phosphorylation of Tyr985 in Ob-Rb that leads to an attenuated leptin signaling (100).

In a case-control study, leptin was measured by ELISA in two independent cohorts of biopsy-proven obese NAFLD patients and healthy-liver controls aimed to evaluate this biomarker as a non-invasive method for NAFLD diagnosis. In both cohorts, leptin was increased with similar serum levels as well as diagnostic performance (AUROC >0.80), suggesting that serum leptin can identify NAFLD without obesity (101). In a meta-analysis, leptin levels were compared between NAFLD *vs* controls, and it was shown that circulating leptin levels were higher in NAFLD patients *vs* controls (standardized mean difference 0.640; 95% CI 0.422, 0.858) and it was also associated with NAFLD severity (102). Leptin is a vital biomarker of NAFLD, and the evaluation of its *in vivo* concentration level is of great significance for NAFLD diagnosis. Therefore, the development of a method for a rapid and sensitive detection of leptin is urgent (103). Based on these observations, these adipokines may be appropriate biomarkers of NAFLD; along these lines, Kim et al. concluded that leptin may be a significant predictor for NAFLD in subjects with weight gain.

In conclusion, leptin may seemingly protect against hepatic steatosis in the initial stages of the disease. Nonetheless, leptin may act as an inflammatory and fibrogenic agent when the disease progresses. Leptin deficiency can lead to hepatic steatosis, whereas excessive leptin can promote hepatitis and fibrosis (18).

### 3.6 Insulin

Insulin is an anabolic hormone that mediates ionic transport and storage of TG in adipose tissue; it also regulates glucose levels and can inhibit lipolysis. In physiological terms, insulin is secreted by pancreatic β-cells after a meal or after hormone release as glucagon or catecholamine. Since the 80s, insulin has been considered a modulator of satiety signals (104); however, insulin is involved in both satiety and hunger signals and depends on the interaction with other peptides and hormones, majorly with leptin. Together, leptin and insulin modulate the expression of proopiomelanocortin inducing appetite sensation; on the other hand, the activation of potassium channels through Phosphoinositide 3-kinases, at the hypothalamus, reduces the appetite sensation (105). Insulin has been related to memory processes implicated in food intake according to their energy content, regulating appetite in relation to previous meals (106); due to this, the administration of nasal insulin has been studied with the aim of decreasing food intake in animal and human models (107).

Hepatic IR describes impaired suppression of hepatic glucose production, which largely accounts for hyperglycemia and glucose intolerance (108). The association of IR and NAFLD is supported strongly by clinical and laboratory data. NAFLD has been shown to be closely associated with insulin resistance, as 70%–80% of obese and diabetic patients have NAFLD (109).

Since IR is one of the major pathways in NAFLD, the appetite and satiety signals modulated by insulin could be impaired in these patients; most pharmacological treatments aim to improve IR and their linked comorbidities in NAFLD patients; therefore, with the improvement of IR, NAFLD and other metabolic markers could improve. The consequence could be a beneficial effect in hunger and satiety modulation, with an impact in energy intake.

**Figure 2:**
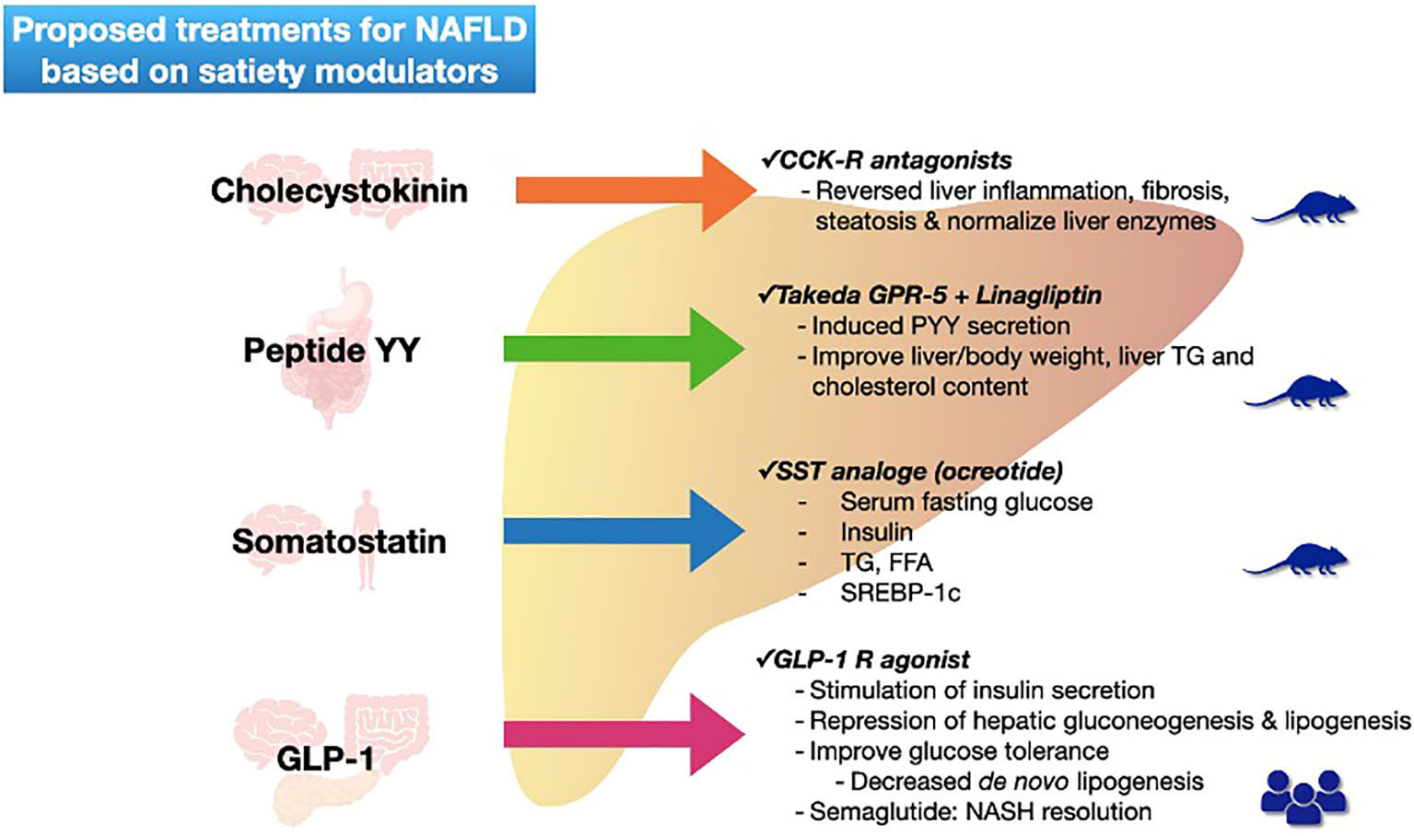
Proposed treatments for NAFLD based on satiety modulators. Figure shows each satiety modulator (with their secretion site) and the actual proposed treatments with action mechanism for NAFLD resolution. Mice figure represents level of evidence (basic), and human figures represent patient studies. NAFLD non-alcoholic fatty liver disease; GLP-1 glucagon -like peptide-1; CCK-R cholecystokinin receptor; GPR G-protein-coupled receptors; PYY peptide YY; TG triglycerides; SST somatostatin; FFA free fatty acids; SREBP sterol regulatory element binding proteins; NASH non-alcoholic steatohepatitis.

Concerning treatment options based on satiety modulators most of the effects have been observed in animal models, only GLP-1 receptor agonists have been studied in patients being semaglutide the most promised treatment (**Figure 2**).

## 4 Concluding remarks

A number of agents that control hunger and food intake by signals on the brain has been identified above. These could be manipulated in order to suggest treatment options for obese NAFLD patients.

The primary goal of NAFLD treatment is weight loss, and if weight is related to energy intake, we might influence the hunger and satiety mechanism to achieve this goal, as this is the beginning of the primary cause of disrupted energy intake. Nowadays, BS is a treatment option for obesity and their comorbidities such as NAFLD. Regarding hunger and satiety signals, BS has been demonstrating diverse effects. The Fxr-Glp-1 axis improves glucose homeostasis through the bile diversion to ileum caused by BS; this has demonstrated to mediate metabolic changes, depending on bile acids bioavailability, which could be an option for obesity and DM treatment, both linked to NAFLD (110). These changes in bile acids have also been associated with improvement of intestinal microbiota and, consequently, with the action of leptin and Ghr (111, 112). On the other hand, the improvement on GLP-1, added to lifestyle changes and emotional support, has been associated with less weight regain after BS (113). As to the abnormal response to Ghr in obese patients, it seems to be reestablished after a Roux-en-Y gastric bypass procedure (114). Although BS has demonstrated beneficial effects on hormones and peptides related to hunger and satiety signals, it is not one of the first line NAFLD treatments; however, its effect on hunger and satiety impairments could improve the clinical metabolic markers, including those related to liver disease, in NAFLD patients undergoing bariatric procedures.

Therefore, we need to address hormones, peptides, cytokines, and metabolites involved in hunger and satiety pathways with the aim of achieving a reduction in hunger sensation and increasing the satiety mechanisms. We might discuss the benefits of the effect of novel therapies focused on antagonism or agonism in receptors of the previously reviewed molecules.

### 4.1 Antagonism in molecules for novel target therapies in obese NAFLD patients

Hunger signaling regulation catches interest in the effects of Ghr and its receptor antagonist on BW loss because the inhibition of the Ghr pathway reduces food intake, body weight, and adiposity through appetite reduction and augmentation of energy expenditure and fat catabolism. Acylated-Ghr might induce IR and promote liver lipid accumulation via a central mechanism located in the hypothalamus. Antagonism in Ghr hormone may allow obese NAFLD patients to reduce the development and persistence of obesity with prolonged periods of diet maintenance. Also, another interesting antagonist mechanism to treat NAFLD is found in the NPY, the expression of which correlates with the liver mass/BW ratio, making it a promising target for therapeutic approaches in adiposity reduction. Knowing the essential role of AgRP, a protein responsible for voracious feeding behavior, and that this peptide can be antagonized with the leptin hormone, we could think of targeting the leptin agonist as a new pharmacological perspective to achieve satiation and the intrinsic modulation of AgRP/NPY neurons (115). The emerging antagonist therapies for NAFLD include MCH and MCH1 receptors. Their effects have been shown in mice, although not in humans, making them potential targets for appetite suppression.

### 4.2 Agonism in molecules for novel target therapies in obese NAFLD patients

Although TH with their metabolites and receptor beta have been proposed as therapeutic targets, evidence related to thyroid function in NAFLD/NASH is still controversial; therefore, further studies are necessary to evaluate these interactions and possible therapeutic effects. GC role in NAFLD development has not been sufficiently analyzed in clinical studies. Even though the high production of GC has been related to fatty acids metabolism and characteristic metabolic abnormalities of NAFLD, their action involves many and different pathways; however, the interplay between GC with cholesterol and fatty acids uptake could be an interventional strategy for this liver disease. Meanwhile, the previously reviewed results suggest that hypothalamic orexin is an essential factor for preventing NASH and associated HCC under obesity; a novel therapy with orexin supplementation might arise from this review. CCK also acts as a modulator of the MCH-1 receptor, making it a double-effect therapy. A CCK agonist has been demonstrated to achieve anti-obesity effects with remarkable efficacy, reducing weight and appetite signals, but also acting like a GLP-1 potentiator when used as adjunct therapy. With regard to gut-derived hormones, Semaglutide is one of the strongest analogues tested nowadays, being superior to placebo, as recent studies have demonstrated with obese and NAFLD patients, preventing the progression to fibrotic stages.

Another gut-derived peptide is PYY; its secretion improves liver steatosis. Insulin sensitivity indicates that inducing the secretion of incretins, such as PYY, may have a therapeutic potential in NAFLD patients. Hepatic glycogenesis, a cause of metabolic abnormalities in NAFLD, is also reduced. STT might promote weight loss and improve the metabolic disorder in NAFLD patients, since octreotides have demonstrated to restore increased TG and FFA levels (94).

Leptin has different roles in NAFLD, according to the disease stage; in initial stages it could act as a protection factor, but when NAFLD progresses, leptin acts as an inflammatory and fibrogenic agent. With these dual roles, leptin could be proposed as treatment for suppressing hunger and food intake, either through a leptin receptor agonist or through an increment of its release. Yet the stage of the disease must be carefully evaluated before proposing this therapy.

As previously seen, insulin and IR are correlated in NAFLD pathophysiology. Among anti-diabetic medications, GLP-1 receptor agonists demonstrate a significant improvement in the hepatic histology. Therefore, their implementation is crucial for NAFLD management (116).

## 5 Conclusion

Despite the lack of a specific molecule for NAFLD treatment, nowadays there are several options that could improve different pathways of this chronic liver disease; however, it has been observed that one of the major challenges in these patients is the maintenance of lifestyle modifications. Therefore, in the near future, antagonist/agonist of hunger and satiety signals could have a key role in patients with improved NAFLD by avoiding weight gain and liver steatosis recurrence.

Clearly, hunger and satiety mechanisms are relevant factors in the spectrum of NAFLD pathophysiology and some of their pathways are interconnected, but since liver damage is multifactorial, any modification in these mechanisms is insufficient to impact the development of NAFLD; however, the knowledge of the implications of hunger and satiety in the metabolic profile, and the interplays between them, and according to the patient’s profile (hormonal, obesity, IR, and comorbidities), we could select a pharmacological approach that involves different pathways with the intention of designing an individualized treatment that encompasses all NAFLD alterations, from microbiome signature to hormone and genetic profile, second messengers’ expression, and inflammation patterns.

As a chronic disease encompassing a wide spectrum of damage, NAFLD is now a public health burden with significant impacts on morbidity and mortality. In relation to hunger and satiety modulators, more research is required to assess the safety and efficacy of treatments based on modifications of these signals, specifically clinical studies.

## Author contributions

Conceptualization: EJ-H, IL-M. Methodology: EJ-H, IL-M. Literature Analysis: AM-R. Writing- original draft preparation: AM-R. Writing-review and editing: IL-M, EJ-H, MU. Supervision: MU. All authors contributed to the article and approved the submitted version.
